# Excess mortality at Christmas due to cardiovascular disease in the HUNT study prospective population-based cohort in Norway

**DOI:** 10.1186/s12889-021-10503-7

**Published:** 2021-03-20

**Authors:** Trine Moholdt, Clifford Afoakwah, Paul Scuffham, Christine F. McDonald, Louise M. Burrell, Simon Stewart

**Affiliations:** 1 Department of Circulation and Medical Imaging, Norwegian University of Science and Technology, Trondheim, Norway; 2 The Women’s Clinic, St.Olav Hospital, Trondheim, Norway; 3 Centre for Applied Health Economics, Griffith University, Nathan, Queensland Australia; 4 Menzies Health Institute Queensland, Griffith University, Southport, Queensland Australia; 5 Department of Respiratory and Sleep Medicine, Austin Health, Institute for Breathing and Sleep, University of Melbourne, Melbourne, Australia; 6 Department of Medicine, Austin Health, University of Melbourne, Melbourne, Australia; 7 Torrens University Australia, South Australia, Wakefield Campus, Adelaide, SA 5000 Australia; 8 University of Glasgow, Glasgow, Scotland UK

**Keywords:** Population cohort, Longitudinal follow-up, Mortality, Cardiovascular disease, Seasonality

## Abstract

**Background:**

Although it is known that winter inclusive of the Christmas holiday period is associated with an increased risk of dying compared to other times of the year, very few studies have specifically examined this phenomenon within a population cohort subject to baseline profiling and prospective follow-up. In such a cohort, we sought to determine the specific characteristics of mortality occuring during the Christmas holidays.

**Methods:**

Baseline profiling and outcome data were derived from a prospective population-based cohort with longitudinal follow-up in Central Norway - the *Trøndelag Health (HUNT) Study.* From 1984 to 1986*,* 88% of the target population comprising 39,273 men and 40,353 women aged 48 ± 18 and 50 ± 18 years, respectively, were profiled. We examined the long-term pattern of mortality to determine the number of excess (all-cause and cause-specific) deaths that occurred during winter overall and, more specifically, the Christmas holidays.

**Results:**

During 33.5 (IQR 17.1–34.4) years follow-up, 19,879 (50.7%) men and 19,316 (49.3%) women died at age-adjusted rate of 5.3 and 4.6 deaths per 1000/annum, respectively. Overall, 1540 (95% CI 43–45 deaths/season) more all-cause deaths occurred in winter (December to February) versus summer (June to August), with 735 (95% CI 20–22 deaths per season) of these cardiovascular-related. December 25th–27th was the deadliest 3-day period of the year; being associated with 138 (95% CI 96–147) and 102 (95% CI 72–132) excess all-cause and cardiovascular-related deaths, respectively. Accordingly, compared to 1st–21st December (equivalent winter conditions), the incidence rate ratio of all-cause mortality increased to 1.22 (95% CI 1.16–1.27) and 1.17 (95% 1.11–1.22) in men and women, respectively, during the next 21 days (Christmas/New Year holidays). All observed differences were highly significant (*P* < 0.001). A less pronounced pattern of mortality due to respiratory illnesses (but not cancer) was also observed.

**Conclusion:**

Beyond a broader pattern of seasonally-linked mortality characterised by excess winter deaths, the deadliest time of year in Central Norway coincides with the Christmas holidays. During this time, the pattern and frequency of cardiovascular-related mortality changes markedly; contrasting with a more stable pattern of cancer-related mortality. Pending confirmation in other populations and climates, further research to determine if these excess deaths are preventable is warranted.

**Supplementary Information:**

The online version contains supplementary material available at 10.1186/s12889-021-10503-7.

## Background

Although age-standardised mortality is typically reported as the number of deaths per 1000 people at risk per annum, deaths are rarely evenly distributed throughout the year. Typically, more deaths due to cardiovascular disease (CVD) occur in winter compared to summer [1]. Paradoxically, seasonal variations in cardiovascular-related mortality are not simply explained by exposure to environmental provocations such as cold temperatures, reduced daylight hours, infections, or increased pollution [2–5]. Rather, they appear to reflect a more complex interplay between the environment and an individual’s physical and psychological condition, their behaviours and the culture/society in which they live [4, 6]. In Scandinavia, for example, an individual-to-societal adaptation to extremely cold temperatures undoubtedly mitigates the cyclic exposure and physiological responses to seasonally driven provocations to cardiovascular health [7].

Previous studies have sought to link clusters of increased mortality to large earthquakes [8] and the FIFA World Cup [9]. Beyond these exceptional events, there is an event that has strong potential to be detrimental to an individual’s cardiovascular health on an annual basis [10, 11]. At Christmas, people around the world engage in potentially stressful social interactions and provocative behaviours they would not normally expose themselves to. In those already at risk of seasonal patterns of mortality (i.e., where Christmas coincides with winter), these factors may act as additional, short-term triggers for a broad range of cardiovascular-related events [12]. A number of studies based on administrative data have previously demonstrated increased rates of mortality [12, 13], hospitalisation [11] and acute myocardial infarction (AMI) in Sweden during the Christmas holidays [10]. Beyond these studies, however, this phenomenon remains poorly characterised [1].

We hypothesised that over and beyond long-term seasonal trends within a population periodically exposed to cold winters, we would find an additional risk of dying over the Christmas holidays. In effect this would represent an increased period of increased mortality within an already high-risk period of the year. We also hypothesised that CVD would be the major contributor to this phenomenon and that we would find sex-specific differences in this regard.

## Methods

### Study context

Norway (population ~ 5.5 million people) has a long tradition of undertaking insightful, longitudinal population cohort studies. This includes the Tromsø Study in Northern Norway [7, 14], and the focus of this report, the Trøndelag Health (HUNT) Study [15]. Although the warm currents of the Gulf Stream moderate its weather, given its northerly latitude, Norway experiences extreme weather conditions. Central Norway’s Köppen Climate Classification subtype is *Continental Subarctic Climate* [16]. The coldest month is January (mean temperature of minus 3 °C) and the warmest month is July (around 13 °C) with a mean annual temperature of 4.8 °C overall. Although Norway enjoys relatively clean air, the winter solstice and darkest days of the year coincide with Christmas.

### Study design

We examined the long-term pattern of mortality within the prospective, longitudinal, population-based HUNT Study cohort living in Central Norway [15, 17]. The present study was approved by the Regional Committee for Ethics in Medical Research (REK-midt, no. 2018/1509).

### Data collection

The original wave of population screening (HUNT1) was undertaken during 1984–1986, with 88% of eligible inhabitants aged ≥20 years in Nord-Trøndelag County recruited. Here, we include the 79,626 men and women who attended a clinical examination and filled out detailed questionnaires about their health and lifestyle [15]. Specifically, data on socio-economic status, perceived levels of health and life satisfaction, lifestyle behaviours, and self-reported cardiovascular health CVD were derived from validated questionnaires [15, 17]. We used a previously developed index of physical activity to categorise levels of leisure-time physical activity [18].

### Study outcomes

The unique personal identification number of all Norwegian citizens allows linkage of each participant’s record in the HUNT Study to information from the national Cause of Death Registry on the timing and primary cause of death. These are classified according to the International Classification of Disease (ICD) – with precise death coding data available until 1st January 2018. Based on the listed causes of death and the pre-specified hypotheses, the main codes of interest were - CVD (i.e., ICD-9: 390–459 and ICD-10: I00-I99t inclusive of the specific codes for coronary artery disease [CAD], acute myocardial infarction [AMI], cerebral infarction, and sudden cardiac death), as well as cancer/malignancy and respiratory disease/illness. Regardless of cause of death coding, data on the occurrence and timing of death were available for the full follow-up period (1984 to 2020). Using these data, the specific focus of this study was calculating (if appropriate) the number of excess deaths (on a crude and adjusted basis) occurring during the key time-points of interest.

### Data analyses

This study conforms to the STROBE guidelines for the reporting of observational studies [19]. Deaths were initially grouped according to whether they occurred in winter (December, January, and February), spring (March, April, and May), summer (June, July, and August) or autumn (September, October. and November). Mortality data were also grouped into 3-day rolling totals to identify potentially more specific periods of increased mortality (including Christmas). Subsequently, three specific 21-day periods were purposefully selected for more granular analyses and comparison – 1) the 21 days in which, on a statistical basis, the least number of all-cause deaths occurred (17th May-6th June); 2) the 21 days of winter preceding Christmas (1st–21st December – selected as the reference period for all comparisons) in which mortality rates were reflective of the broader winter period and; 3) the subequent 21 days inclusive of the Christmas holiday period (22nd December-11th January) in which mortality rates were elevated above the winter average. The main outcome variable is the counts of deaths per day while the main exposure variable is the time (for example, Christmas or winter period). We modelled excess mortality by adjusting for baseline characteristic such as sex, age at death, month, and annual trends. The number of lower/excess deaths per period was then estimated using the ordinary least squares (OLS) method. A Poisson approach was then used to estimate the increased/decreased risk of mortality (incidence rate ratio [IRR] with 95% CI’s) due to exposure to the Christmas holiday period. Using the variables summarised in Table 1, we generated adjusted hazard ratios (HR) for all-cause mortality for the entire cohort during the median study period of 33.5 (IQR 17.1 to 34.4) years follow-up using a Cox-Proportional Hazard model (entry model using only those cases with full profiling data). These same methods (Cox-Proportional Hazard models) were used to directly compare the correlates of dying in – a) the first 21 days of winter (December 1st to 21st) versus the lowest 21-day period of deaths during the rest of the year (May 17th to June 6th) in 2894 participants who died during this combined 42-day period) and b) December 22nd to January 11th (21 days inclusive of Christmas/New Year holidays) versus the preceding 21 days (December 1st to 21st) on a sex-specific basis. All analyses were performed using SPSS v26.0 and STATA v13. Statistical significance was accepted at a 2-sided alpha of *P* < .05.

**Table 1:** Baseline characteristics according to survival status

	Total (***n*** = 79,626)	Alive (***n*** = 40,431)	Dead (***n*** = 39,195)
**Demographic Profile**			
Women, %	40,353 (50.7)	21,037 (52.1)	19,316 (47.9)
Age Groups, %			
Aged < 35 years	22, 208 (27.9)	20, 739 (50.4)	1469 (3.7%)
Aged 35–44 years	15,556 (19.5)	12,602 (31.2)	2954 (7.5%)
Aged 45–64 years	24,874 (31.2)	7063 (17.5)	17,811 (45.4%)
Aged 65+ years	16,988 (21.3)	27 (0.001)	16,961 (43.3%)
Mean age at baseline (years)	49.1 ± 18.0	35.7 ± 9.6	62.9 ± 13.6
Mean age at end follow-up (years)	74.9 ± 11.6	70.1 ± 11.4	79.9 ± 9.6
Married, % (*n* = 76,775)	52,709 (66.6)	26,532 (69.5)	26,177 (67.8)
≤ 9 years education, % (*n* = 61,240)	32,928 (53.9)	9082 (29.9)	23,886 (77.4)
Employment status, % (*n* = 76,870)			
Full-time employment	32,333 (42.1)	21,585 (66.8)	10,748 (28.0)
Part-time employment/housework	21,381 (27.8)	13,219 (34.4)	8162 (21.2%)
Non-employed/ retired	23,156 (30.1)	3623 (15.6)	19,533 (50.8)
**Health Status**			
Life Satisfaction, % (*n* = 75,815)			
*Dissatisfied (Quite to Extremely)*	2005 (2.6)	630 (1.7)	1375 (3.6)
*Satisfied (Quite to Extremely)*	62,342 (82.2)	32,967 (86.6)	29,375 (77.9)
General Health Status, % (*n* = 76,863)			
*Bad*	2023 (2.6)	202 (0.5)	1821 (4.7)
*Poor*	18,752 (24.4)	4615 (12.0)	14,317 (36.7)
*Good*	44,215 (57.5)	24,411 (63.6)	19,804 (51.5)
*Very Good*	11,873 (15.4)	9165 (23.9)	2708 (7.0)
Physical Activity Status, % (n=57,212)			
*Inactive*	27,145 (47.4)	13,157 (45.1)	13,988 (49.9)
*Low*	18,730 (32.7%)	9728 (33.3)	9002 (32.1)
*Moderate*	8283 (14.5)	4951 (17.0)	3332 (11.9)
*High*	3054 (5.3)	1362 (4.7)	1692 (6.0)
Alcohol intake, % (*n* = 61,520)			
*4 or less drinks in 14 days*	50,376 (81.9)	27,143 (88.3)	23,233 (76.6)
*5 or more drinks in 14 days*	3608 (5.9)	1685 (5.5)	1923 (5.1)
*Abstains*	7536 (12.2)	1899 (6.2)	5637 (18.3)
Current smoker, % (*n* = 60,421)	20,667 (34.2)	10,885 (36.7)	9782 (32.9)
Mean BMI kg/m^2^ (*n* = 74,330)	25. ±3.9	24.2 ± 3.4	26.2 ± 4.1
Mean heart rate, bpm (*n* = 74,906)	74.9 ± 12.6	73.8 ± 11.9	76.0 ± 13.1
Mean BP, mmHg (*n* = 74,832)			
Systolic BP/	139 ± 23.5 /	127 ± 15.2 /	150 ± 25.0 /
Diastolic BP	84.6 ± 15.2	80.9 ± 15.2	88.5 ± 11.7
Angina pectoris (%) (*n* = 76,742)	3450 (4.5)	113 (0.3)	3337 (8.7)
AMI, % (*n* = 76, 723)	1986 (2.6)	39 (0.1)	1947 (5.1)
Stroke, % (*n* = 76,794)	1412 (1.8)	59 (0.2)	1353 (3.5)

## Results

### Cohort characteristics

The study cohort comprised 40,353 women (50.1%) and 39,273 men aged 50 ± 18 and 48 ± 18 years, respectively. Two-thirds were married and just over half had < 10 years of formal education. Most participants reported generally positive health and life-satisfaction levels. Alternatively, many had relatively high levels of risk for CVD and other chronic diseases, including elevated baseline levels of blood pressure (BP) and smoking combined with relatively high levels of sedentary behaviours and overweight status (Table 1).

### All-cause mortality

**Fig. 1:**
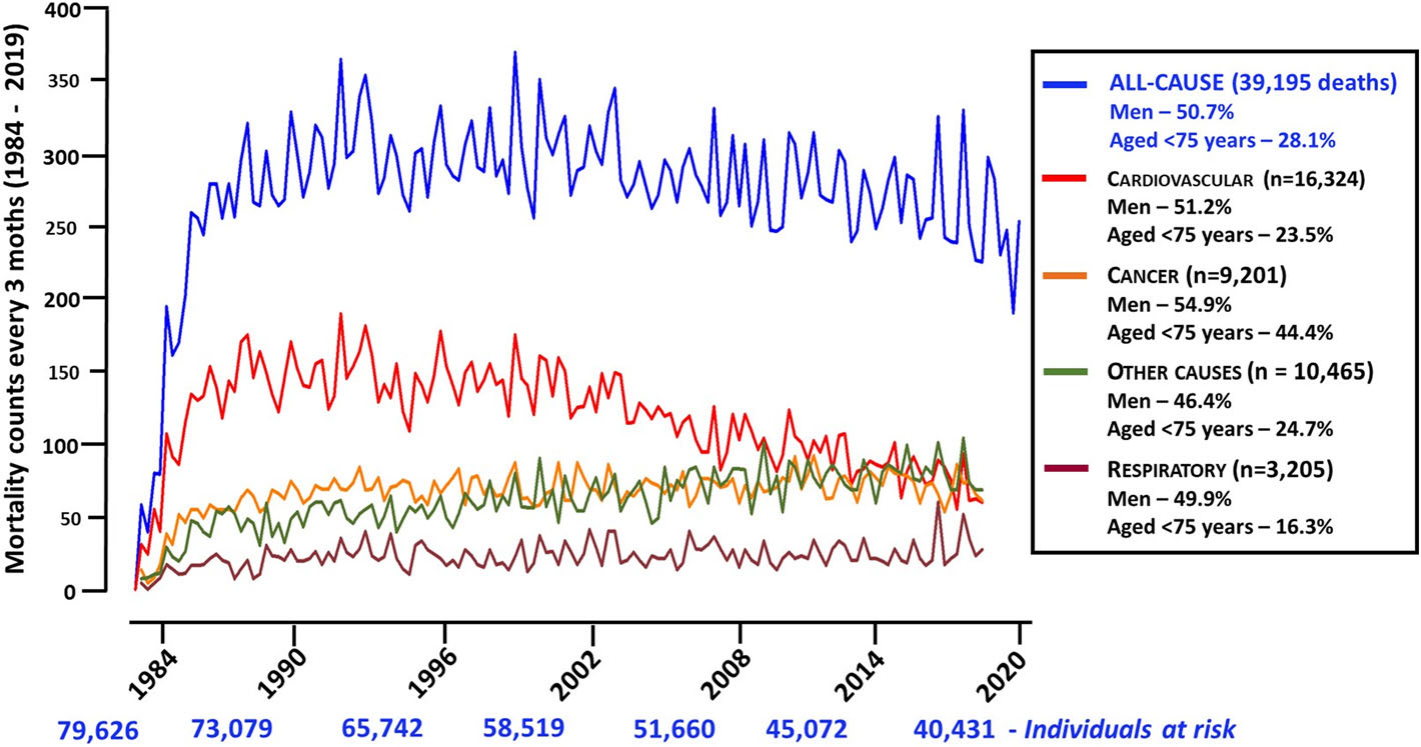
Fluctuating Patterns of Mortality. Total all-cause and cause-specific death counts were plotted in 3-monthly intervals (synchronised to each distinctive season) over the entire 35-year study period

During the 35-year study period, there were 39,195 deaths (49.2%) comprising 19,879 (50.7%) men and 19,316 (49.3%) women. As shown in Fig. 1, these deaths were not evenly distributed over time. Age-adjusted mortality was slightly higher in men compared to women (5.3 and 4.6 deaths per 1000/annum, respectively); rising from 1.6 to 224 deaths and from 1.1 to 183 deaths per 1000/annum in men and women initially aged < 30 years and > 80 years, respectively. An increased risk of all-cause mortality (*P* < .001 for all comparisons unless indicated) was correlated with advancing age (adjusted HR 1.11, 95% CI 1.11–1.12 per year), male sex (1.59, 1.55–1.64 versus women), lower education (1.15, 1.11–1.18 for ≤9 years education versus rest), greater unhappiness (1.30, 1.21–1.39 for any degree of life dissatisfacton versus rest), being divorced/separated (1.15, 1.06–1.20 versus unmarried), obesity (1.13, 1.09–1.18), being a current smoker (1.89, 1.79–1.91 versus rest), excessive alcohol intake (1.09, 1.02–1.16 for > 10 drinks in 14-days versus abstinence; *P* = .017), an elevated heart rate (1.03, 1.02–1.03 per 5 beats/min), higher systolic (1.02, 1.02–1.03 per 5 mmHg) and diastolic BP (1.01, 1.00–1.02 per 5 mmHg), as well as a self-reported history of AMI (1.65, 1.55–1.76), angina pectoris (1.26, 1.20–1.33) and stroke/cerebral event (1.48, 1.36–1.60). Alternatively, being married (adjusted HR 0.80, 95% CI 0.77–0.83 versus unmarried), better self-reported general health (0.76, 0.74–0.79 for good/very good versus rest), mild alcohol intake (0.94, 0.91–0.97 1–4 drinks in 14-days versus abstinence) and greater levels of exercise (0.89, 0.86–0.92 for moderate to high adherence to recommended exercise versus rest) were associated with a reduced risk of all-cause mortality.

### Specific causes of death

The three most common causes of death in men and women were CVD (8355 [43.6%] and 7969 deaths [43.0%], respectively), cancer (5051 [26.4%] and 4150 deaths [22.4%]), and respiratory disease/illness (1599 [8.3%] and 1606 [8.7%] deaths). Collectively, these accounted for 78 and 74% of all deaths in men and women, respectively. Other causes of death included endocrine disorders (1343 [3.4%]), psychiatric disorders (1118 [2.9%]) and external factors including motor vehicle accidents and violence (951 [2.5%]). Consistent with all-cause mortality, there were marked fluctuations (with clear peaks and troughs) in those deaths attributable to CVD and respiratory disease.

### Seasonal patterns of mortality

**Fig. 2:**
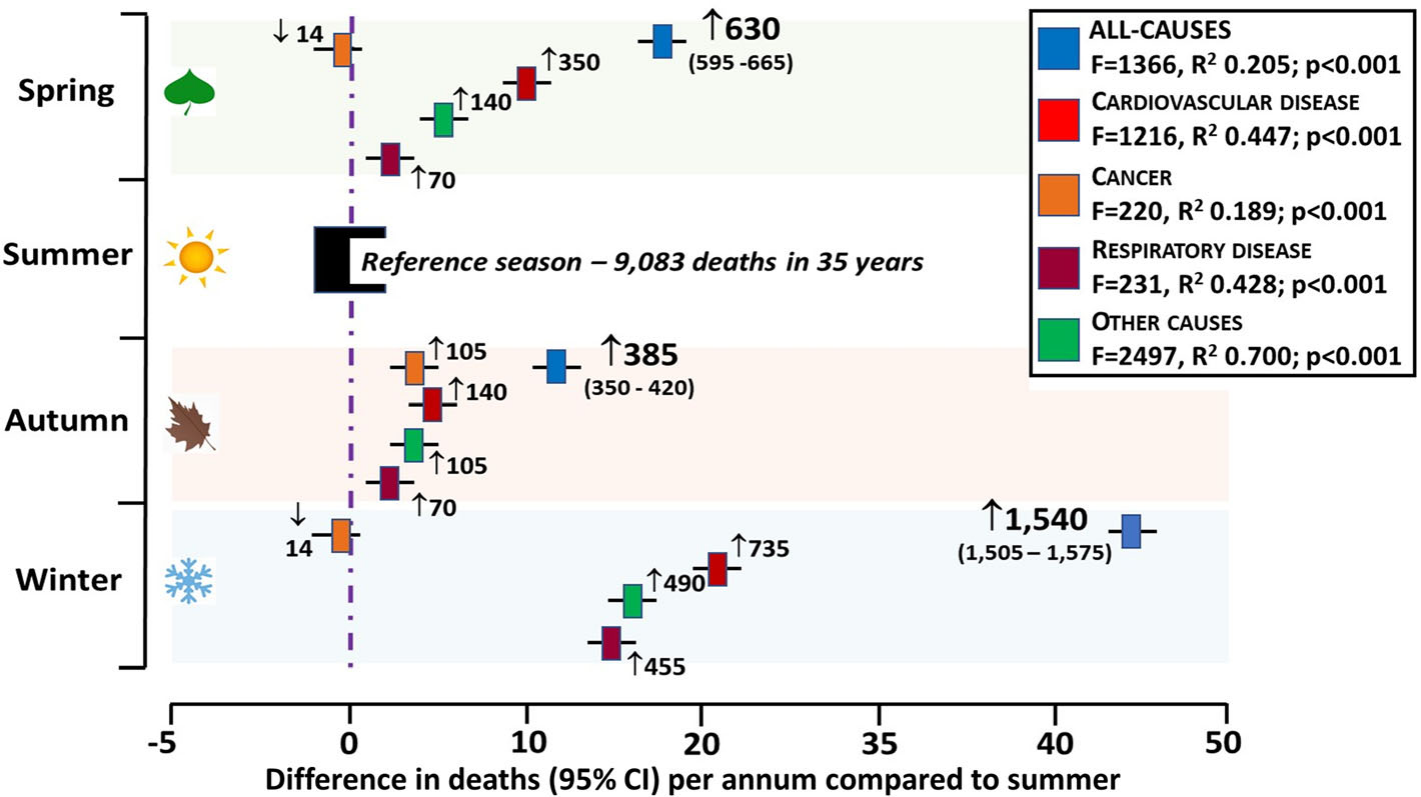
Seasonal Comparisons of Mortality. The adjusted, annual number of deaths (error bars show 95% CI) occurring in spring, autumn, and winter are plotted above and below the reference (low mortality) season of summer for - all-cause mortality (blue symbols) and those related to cancer (orange), CVD (red), respiratory disease (green) and other causes (brown). The total difference in deaths for all-causes (with 95% CI) and specific causes over the entire 35-years are also shown adjacent to each symbol

On an absolute basis, 1707 more deaths occurred in winter (10,790 [27.5%]) compared to summer (9083 [23.2%]) during the 35-year study period. The differential between cardiovascular- and respiratory-related mortality occurring in winter (4446 [27.4%] and 1037 [32.4%] deaths) versus summer (3832 [23.5%] and 661 [20.6%] deaths) contributed to 59% (1010 deaths) of the observed variance between winter and summer. Although a more stable pattern of mortality was observed in spring (9900 [25.3%] deaths) and autumn (9442 [24.0%] deaths), a seasonal pattern was still evident. On adjusted basis, each winter there were 44 (95% CI 43–45/annum) more deaths when compared to the equivalent 3 months of summer. The main contributors to the excess deaths occurring in winter were CVD (21, 95% CI 20–22 deaths/annum), respiratory disease (13, 95% CI 13–14 deaths/annum) and other miscellaneous conditions (14, 95% CI 13–14 deaths/annum). Alternatively, as indicated by Fig. 2, over the entire 35-year study period, cancer-related deaths occurred at a far more stable, seasonal rate (the absolute difference between winter versus summer-being 10 deaths).

### The Christmas holiday effect

**Fig. 3:**
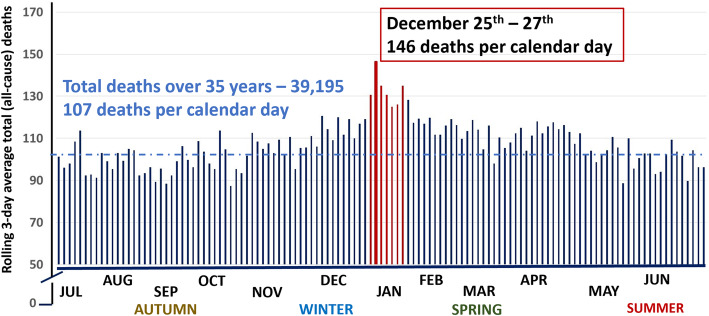
3-Day Mortality Across the Calendar Year. This graph plots the 3-day, rolling average of all-cause deaths occurring during the entire 35-year study period, starting with the calendar days of 1st – 3rd July and ending in the 28th – 30th June. The Christmas period of increased mortality is highlighted in red

**Fig. 4:**
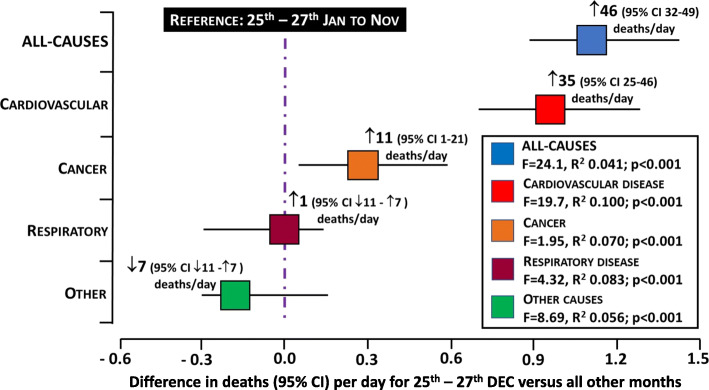
Excess Christmas Mortality. The adjusted, annual number of deaths (error bars show 95% CI) occurring during the 3-day period 25th–27th December are plotted against the reference period (deaths occurring during 25th–27th day of every other calendar month) for - all-cause mortality (blue symbols) and those related to cancer (orange), CVD (red), respiratory disease (green) and other causes (brown). The total difference in deaths (with 95% CI) for all-causes and specific causes over the entire 35-years are also shown above each symbol

Regardless of the season, accumulative 3-day mortality consistently fluctuated between 90 and 110 deaths, apart from a clear increase in mortality commencing on the 22nd of December. The subsequent 3-day period over Christmas was the deadliest of the year (Fig. 3) with 439 all-cause deaths occurring on 25th – 27th December. This was not a random phenomenon and was largely driven by an increase in cardiovascular-related and, to a lesser extent, cancer-related deaths (Fig. 4). On an adjusted basis, over the entire 35-year study period, an additional 138 (95% CI, 114–159) more all-cause deaths occurred during this specific 3-day period compared to those same calendar days during the rest of the year. CVD (an extra 105 [95% CI 75–138] deaths per day) was the main contributor to this phenomenon. This elevated mortality rate persisted until early January. During the 21 days from the 22nd of December, there were 2679 deaths (51.1% women) compared to 2351 deaths (49% women) during the preceding 21 days versus 2016 deaths (49.6% women) during the lowest 21 days of mortality May 17th through June 6th.

Compared to the already elevated levels of mortality observed during the first 21 days of December/winter, over the 35-years study period, on an adjusted basis, there were 28 (95% CI 21–35) more deaths per day during the subsequent Christmas/New Year period. The major contributors to this phenomenon were CVD and to lesser extent, cancer, and other causes – Supplementary Figure S1. When compared to the preceding 21 days, the Christmas period was also notable in respect to within and between differences among men and women in respect to fatal AMI (78 versus 16 more deaths, respectively), strokes (13 fewer versus 32 more deaths) and heart failure (1 more versus 12 more deaths). Similarly, in men and women, the number of cancer- (18 and 29 more deaths, respectively) and respiratory-related (19 and 33 more deaths, respectively) deaths also increased.

### Winter and Christmas vulnerability

Overall, except for cancer-related mortality (both sexes) and respiratory disease in men, compared to the first 21 days of December/winter, the risk of dying in the late spring/early summer period of 17th May to 6th June was significantly lower - Supplementary Figure S2. Alternatively, except for an increased risk of dying from respiratory illnesses/disease among women, men had a higher risk of dying over the equivalent 21-day Christmas period; the major contributor to this increased mortality risk (from 6 to 22% higher overall) being CVD - Supplementary Figure S3.

Beyond advancing age, a combination of baseline demographic, health perceptions and clinical factors were independently correlated with dying during – 1) late spring/early summer (May 17th to June 6th) versus early winter (Dec 1st – 21st), and then 2) early winter (Dec 1st – 21st) versus the Christmas holiday period (Dec 22nd – Jan 11th). Whilst these factors were broadly similar for both sexes, including a 30% reduced risk during the Christmas holidays associated with being married at baseline, there were some notable differences. For example, consistent with an excess number of strokes among women, but not men, during the Christmas holidays, a pre-existing history of stroke conferred a 2-fold risk of dying during this period among women. Educational status among women also appeared to modulate the additional risk of dying during this period – see Table 2.

**Table 2:** Correlates of All-Cause Mortality at Key Periods of the Year

	Dec 1st – 21st (Winter) versus May 17th – June 6th (Summer)				Dec 22nd – Jan 11th (Christmas/New Year) versus Dec 1st – 21st (Winter/Pre-Christmas)			
	Men (***n*** = 1543)	***P***	Women (***n*** = 1351)	***P***	Men (***n*** = 1636)	***P***	Women (***n*** = 1424)	***P***
**Demographic profile, adjusted HR (95% CI)**								
Age at baseline (per year)	1.06 (1.05–1.07)	.001	1.05 (1.04–1.06)	.001	1.06 (1.05–1.07)	.001	1.05 (1.04–1.06)	.001
≤ 9 years education vs. rest	0.92 (0.72–1.78)	.362	0.97 (0.78–1.21)	.804	1.05 (0.90–1.15)	.519	1.25 (1.03–1.52)	.026
Married vs. rest	0.93 (0.56–1.49)	.769	0.98 (0.55–1.69)	.903	0.71 (0.59–0.86)	.001	0.70 (0.54–91)	.001
**Well-being, adjusted HR (95% CI)**								
Good/V. good physical health vs. rest	0.75 (0.64–0.87)	.001	0.82 (0.70–0.95)	.008	0.79 (0.68–0.92)	.002	0.82 (0.70–0.95)	.009
Life dissatisfaction vs. rest	1.65 (1.23–3.23)	.004	1.52 (1.03–2.25)	.036	0.76 (0.50–1.15)	.194	1.13 (0.77–1.66)	.530
**Medical History, adjusted HR (95% CI)**								
Angina pectoris vs. rest	1.26 (0.98–1.63)	.074	1.57 (1.19–2.08)	.002	1.39 (1.06–1.80)	.016	1.99 (1.05–1.84)	.021
Acute myocardial infarction vs. rest	1.52 (1.11–2.07)	.008	1.64 (0.92–2.91)	.092	1.45 (1.06–1.99)	.021	2.41 (1.46–3.80)	.001
Stroke vs. rest	1.23 (0.78–1.94)	.371	1.50 (0.87–2.58)	.142	1.25 (0.78–1.97)	.326	2.01 (1.28–3.17)	.002
**Lifestyle, adjusted HR (95% CI)**								
Current smoker vs. rest	1.28 (1.06–1.53)	.009	1.24 (1.04–1.49)	.018	1.39 (1.16–1.66)	.001	1.51 (1.26–1.82)	.001
**Vital Signs, adjusted HR (95% CI)**								
Heart rate (per 5 beats/minute)	1.03 (1.01–1.06)	.011	1.02 (0.98–1.05)	.360	1.03 (1.00–06)	.045	1.02 (0.99–1.95)	.152
Systolic BP (per 5 mm/Hg)	1.04 (1.02–1.07)	.001	1.04 (1.02–06)	.001	1.03 (1.01–05)	.002	1.03 (1.00–03)	.019
Diastolic BP (per 5 mm/Hg)	0.98 (0.94–1.03)	.445	1.07 (1.03–09)	.001	0.99 (0.96–1.04)	.822	0.96 (0.92–1.01)	0.087

### Sensitivity analyses

We conducted sensitivity analyses by estimating four different models to test if the phenomenon of Christmas-related excess mortality is a reliable and consistent observation. All four models supported the findings of a significant increase in mortality over the Christmas period – Supplementary Table S1.

## Discussion

We investigated the seasonal pattern of mortality within the HUNT Study cohort living in Central Norway. This population cohort is regarded as representative for the Norwegian population as a whole, except for a lower proportion of non-whites and the absence of large cities. Our analyses revealed a striking long-term difference in mortality occurring in winter compared to summer. CVD accounted for half of this seasonality. Although not the coldest, December proved to be the deadliest month, with 22 more people dying each year compared to June. Overall, the 3-day period of 25th–27th December was revealed to be the deadliest time of the year with CVD the major contributor. Critically, both the frequency and cause of death in men and women appeared to change over the Christmas period. Compared to the same pre-Christmas/wintery period, men were 22 and 17% more likely to die from all-causes and CVD (particularly AMI), respectively. In women, the equivalent risk increases were 17 and 15%, with the contribution of CVD (particularly stroke) even more prominent. Although previous studies have also identified a specific Christmas effect on mortality [10–13, 20], we are unaware of any studies and findings equivalent to those reported here.

There is pre-existing evidence to support the hypothesis that Christmas can be harmful to some individuals. A study of the overall pattern of mortality in the US during 1973–2001 revealed a “holiday effect” during Christmas, with ~ 5% excess deaths, after adjustment for the winter season [12]. Similarly, data from a nationwide coronary care unit registry in Sweden revealed a 15% increase in AMI cases during the Christmas holidays [10]. A higher risk of 30-day mortality or readmission among those hospitalised at Christmas in Ontario, Canada has also been found [11]. From a Southern Hemisphere perspective there is both supportive [13] and contrary evidence [21] of an equivalent phenomenon occurring in summer conditions. Overall, our population-based data, suggest that like the US [12], there is an increased risk of dying at Christmas in Norway. This likely applies to similar regions across Europe. To put this phenomenon into perspective, if the same pattern of excess deaths at Christmas had occurred within the entire population of Norway (a minimum of 3 million adults alive in 1980) on an age- and sex-specific basis, there would have been more than 11,000 excess deaths (around 350 more per annum) over the Christmas holidays alone in the past 30 years.

In the (understandable) absence of prospective studies, it is challenging to delineate between the overall impact of winter and a Christmas-specific effect. As shown by the Tromsø Study [14], there is evidence of winter peaks in blood pressure, heart rate, body weight, total cholesterol, and overall CVD risk. Seasonal variation in physical activity may also be an important consideration for cardiovascular-related mortality [22]. Aerobic exercise, especially with high intensity, can acutely lower systolic BP in the hours following exercise [23].

As in many parts of the world, life in Central Norway during the Christmas holiday period is characterised by festive celebrations, travel away from home/central services, and reduced health services. This typically begins in early December and peaks (regardless of public holidays and weekends) during the week of December 23rd to 31st (New Year’s Eve) with concurrent public holidays on December 25th and 26th. Reduced access to follow-up health care was noted to contribute to 26 excess deaths (and 188 hospital readmissions) per 100,000 patients in Canada during the Christmas holidays [11]. However, this phenomenon does not fully explain the size of the phenomenon we observed within our cohort and the contributory reasons are likely to be multifactorial. Consuming a high-fat diet for only 3 days exacerbates insulin resistance and glycolipid metabolism disorders in men with obesity [24]. Even among healthy men, decreasing physical activity for 1–3 weeks decreases insulin sensitivity and attenuates postprandial lipid metabolism [25]. Vascular stiffness, due to impaired endothelial function of the conduit vessels, is an important factor in the development of hypertension and an independent risk factor for a fatal cardiovascular event [25]. After a high-fat meal, which is typically consumed during Christmas in Norway, endothelial function decreases substantially postprandially [26]. The potential negative impact of increased emotional stress associated with dealing with loneliness and family tensions [27] with the potential for seasonally triggered depression [28], also cannot be ignored. As suggested by our sex-specific findings, any, or all of these “stressors” may affect men and women differently. For example, it has been demonstrated that diabetes, high-density lipoprotein levels and triglyceride levels have more impact on cardiovascular health of women compared to men [29]. The emerging literature around Tako-tsubo cardiomyopathy with a predominance of women affected [30] is notable when considering the small, but intriguing, increase in deaths due to heart failure in women, but not men, at Christmas.

Unfortunately, in the absence of specific interventions, expert clinical guidelines rarely mention or address seasonality. We are currently conducting a randomized trial to address seasonal patterns of hospitalization in 300 vulnerable individuals with chronic heart disease in Melbourne, Australia. Beyond ensuring appropriate vaccination against influenza [31], there is a strong justification for more proactive screening and management of high-risk patients by general practitioners leading up to Christmas. The identification of educational levels in women and marriage status as modifying mortality risk in both sexes, reinforces the importance of considering health literacy and the emotional well-being of individuals leading up to provocative times of the year. Promotion of a healthy lifestyle should occur all year round [32], but should perhaps be highlighted and re-emphasized in the lead-up to Christmas: a time of excessive indulgence of all kinds with potentially tragic consequences. The current COVID-19 pandemic both directly (via residual cardio-pulmonary impairment post-infection [33]) and indirectly (via its negative effects on emotional and psychological well-being, patterns of social interaction, seeking care for pre-existing chronic conditions and reduced exercise levels), has further potential to exacerbate Christmas mortality [34].

### Study limitations

To robustly test our primary hypothesis, we examined patterns of long-term mortality within the HUNT cohort [15, 17] in Central Norway. Although this is a well-characterised population, the pattern of risk and subsequent health outcomes in this semi-rural population may not be reflective of the broader Norwegian population or that of Western Europe. Nor was the study specifically designed to examine the issue of seasonal patterns of disease. As previously noted, Norway has a distinctive climate and culture, and these specific conditions may have contributed to our specific findings. Hence, there is a need to validate these findings in other population cohorts with equivalent data. To maintain the size of outcome data for analyses, we relied upon baseline profiling of the original cohort and mortality outcomes. For many individuals there may be multiple contributing causes of death, so any findings from cause-specific mortality data should be interpreted with some caution. The administrative timing of reported deaths (particularly over the Christmas period) may also be disrupted during holiday periods. To date, we have yet to examine the association between observed changes in risk profiles over time with seasonal patterns of mortality. Nor have we confirmed if the same pattern of seasonality and increased risk of death at Christmas is reflected in the pattern of hospital admissions. We have plans to address these limitations. However, we will not be able to ascertain the quality of care and extent of outpatient follow-up at key times such as Christmas and the New Year period. However, the timing of death (unless a sudden cardiac death) is not indicative of exactly when a person becomes unwell and/or is admitted to hospital [11]. Moreover, we do not have specific data on seasonal changes in risk behaviours (e.g. increased alcohol and food intake) to correlate with the subsequent timing and trajectory of illness and death. Finally, we examined the pattern of mortality on a historical basis, during which time, significant changes in the pattern of life-style behaviours and public health measures have occurred.

## Conclusions

During long-term follow-up of the HUNT population cohort, there was a distinctive pattern of a seasonal increase in mortality during winter when compared to summer months. Over and above this broad pattern, a distinctive pattern of excess mortality predominantly, but not exclusively linked to CVD, was evident over the Christmas holiday period. The number of excess deaths over Christmas was substantial.

## Supplementary Information

**Additional file 1: Figure S1.** 21-day Pattern of Mortality – Pre-Christmas versus Summer Low and Christmas Holiday Period. **Figure S2.** Sex-Specific Risk of Mortality – Pre-Christmas versus Summer Low Period. **Figure S3.** Sex-Specific Risk of Mortality – Pre-Christmas versus Christmas Holiday Period. **Table S1.** Sensitivity analyses.

## Data Availability

The Trøndelag Health Study has invited persons aged 13–100 years to four surveys between 1984 and 2019. Comprehensive data from more than 140,000 persons having participated at least once and biological material from 78,000 persons are collected. The data are stored in HUNT databank and biological material in HUNT biobank. HUNT Research Centre has permission from the Norwegian Data Inspectorate to store and handle these data. The key identification in the data base is the personal identification number given to all Norwegians at birth or immigration, whilst de-identified data are sent to researchers upon approval of a research protocol by the Regional Ethical Committee and HUNT Research Centre. To protect participants’ privacy, HUNT Research Centre aims to limit storage of data outside HUNT databank, and cannot deposit data in open repositories. HUNT databank has precise information on all data exported to different projects and are able to reproduce these on request. There are no restrictions regarding data export given approval of applications to HUNT Research Centre. For more information see: http://www.ntnu.edu/hunt/data

## Abbreviations

AMI: Acute myocardial infarction
BP: Blood pressure
BMI: Body mass index
CAD: Coronary artery disease
CVD: Cardiovascular disease
HUNT: Trøndelag Health Study
OLS: Ordinary least squares
