# Supplementary material for: Excess mortality at Christmas due to cardiovascular disease in the HUNT study prospective population-based cohort in Norway
Source: BMC Public Health. 2021 Mar 20;21:549. doi: 10.1186/s12889-021-10503-7 (PMC7980726; doi:10.1186/s12889-021-10503-7)
Supplement: Supplementary file 1 — Additional file 1: Figure S1. 21-day Pattern of Mortality – Pre-Christmas versus Summer Low and Christmas Holiday Period. Figure S2. Sex-Specific Risk of Mortality – Pre-Christmas versus Summer Low Period. Figure S3. Sex-Specific Risk of Mortality – Pre-Christmas versus Christmas Holiday Period. Table S1. Sensitivity analyses. [file 12889_2021_10503_MOESM1_ESM.docx]

## Supplementary Information

**Excess mortality at Christmas due to cardiovascular disease in the HUNT Study prospective population-based cohort in Norway**

Authors

Trine Moholdt, PhD ^a,b^

Clifford Afoakwah, PhD ^c^

Paul Scuffham, PhD ^c,d^

Christine F McDonald, PhD ^e^

Louise M Burrell, PhD ^f^

Simon Stewart, PhD ^g,h^

**Authors Affiliations**

Department of Circulation and Medical Imaging, Norwegian University of Science and Technology, Trondheim, Norway ^a^

The Women’s Clinic, St.Olav Hospital, Trondheim, Norway ^b^

Centre for Applied Health Economics, Griffith University, Nathan, Queensland, Australia ^c^

Menzies Health Institute Queensland, Griffith University, Southport, Queensland, Australia ^d^

Department of Respiratory and Sleep Medicine, Austin Health, Institute for Breathing and Sleep, University of Melbourne, Melbourne, Australia ^e^

Department of Medicine, Austin Health, University of Melbourne, Melbourne, Australia ^f^

Torrens University Australia, Adelaide, South Australia, Australia ^g^

University of Glasgow, Glasgow, Scotland, United Kingdom ^h^

**Corresponding Author**

Professor Simon Stewart FESC FHFA

NHMRC Senior Principal Research Fellow

Torrens University Australia

Wakefield Campus, Adelaide, SA, 5000, Australia

Email: [simon.stewart@laureate.edu.au](mailto:simon.stewart@laureate.edu.au)

https://orcid.org/0000-0001-9032-8998

**Figure S1 21-day Pattern of Mortality – Pre-Christmas versus Summer Low and Christmas Holiday Period**

**
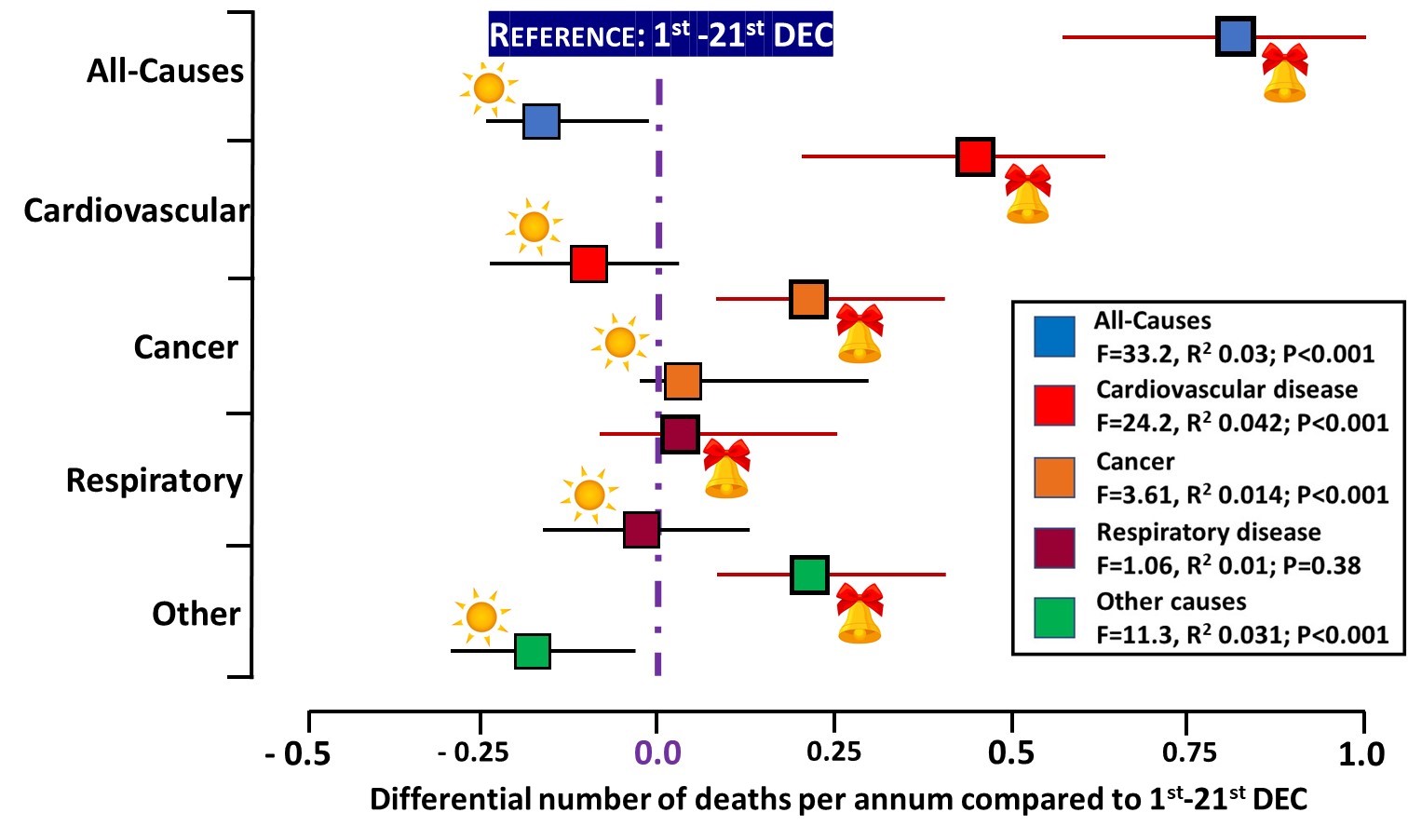
**

**Legend:**  The number of deaths occurring during May 17^th^ – June 6^th^ (sun symbol) and 22^nd^ December to 11^th^ January (Christmas symbols) each year above and below the reference 21-day period 1^st^ – 21st December are shown for all-cause and cause-specific mortality.

**Figure S2 Sex-Specific Risk of Mortality – Pre-Christmas versus Summer Low Period**

**
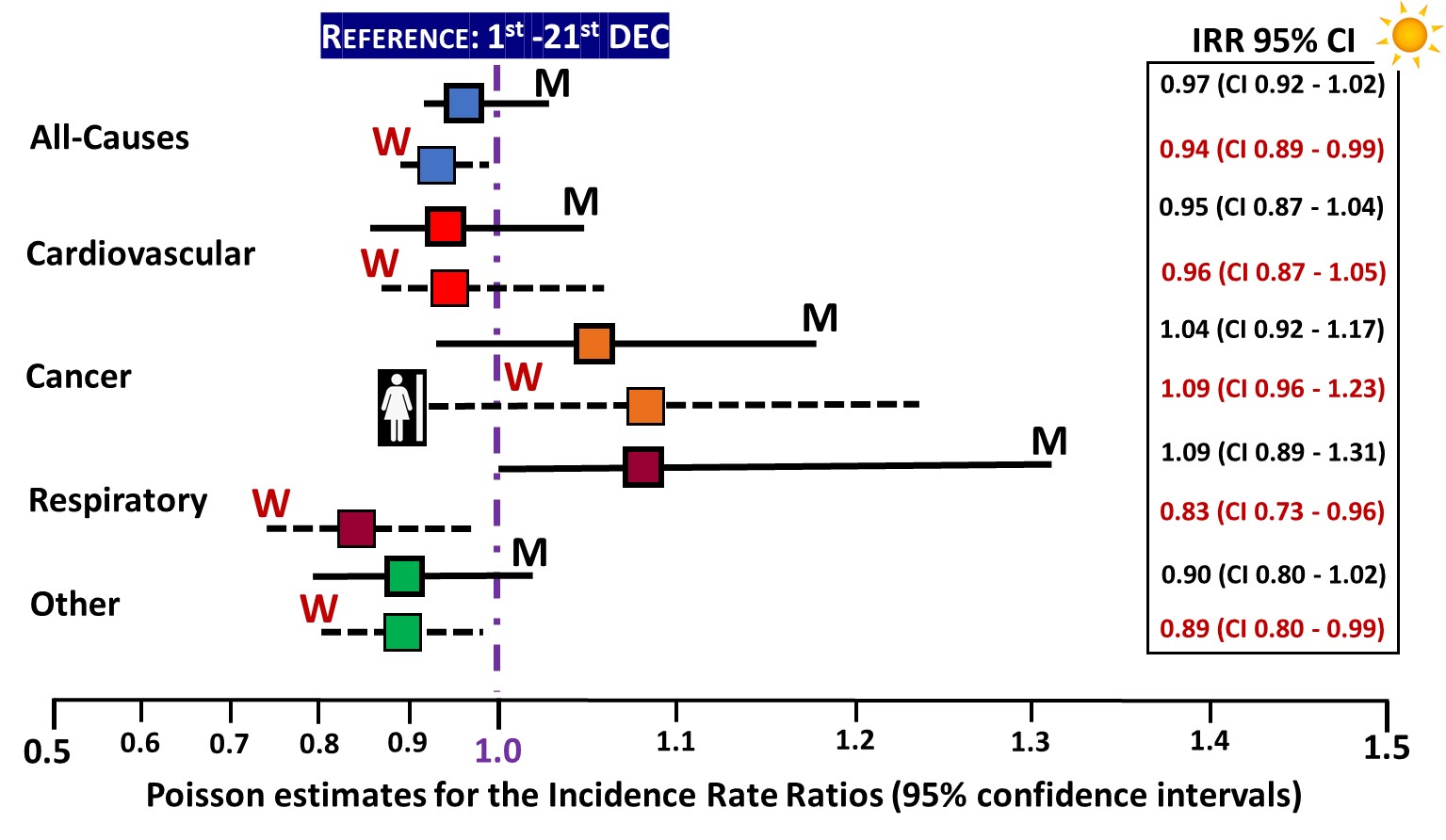
**

**Legend:**  Sex-specific differences (blue figure men and pink figure women) in the incidence of daily mortality (all-cause and cause-specific) during May 17^th^ – June 6^th^ compared to the reference 21-day period 1^st^ – 21st December. M=men (IRR and 95% CI in black) and W=women (IRR and 95% CI in red).

**Figure S3 Sex-Specific Risk of Mortality – Pre-Christmas versus Christmas Holiday Period**

**
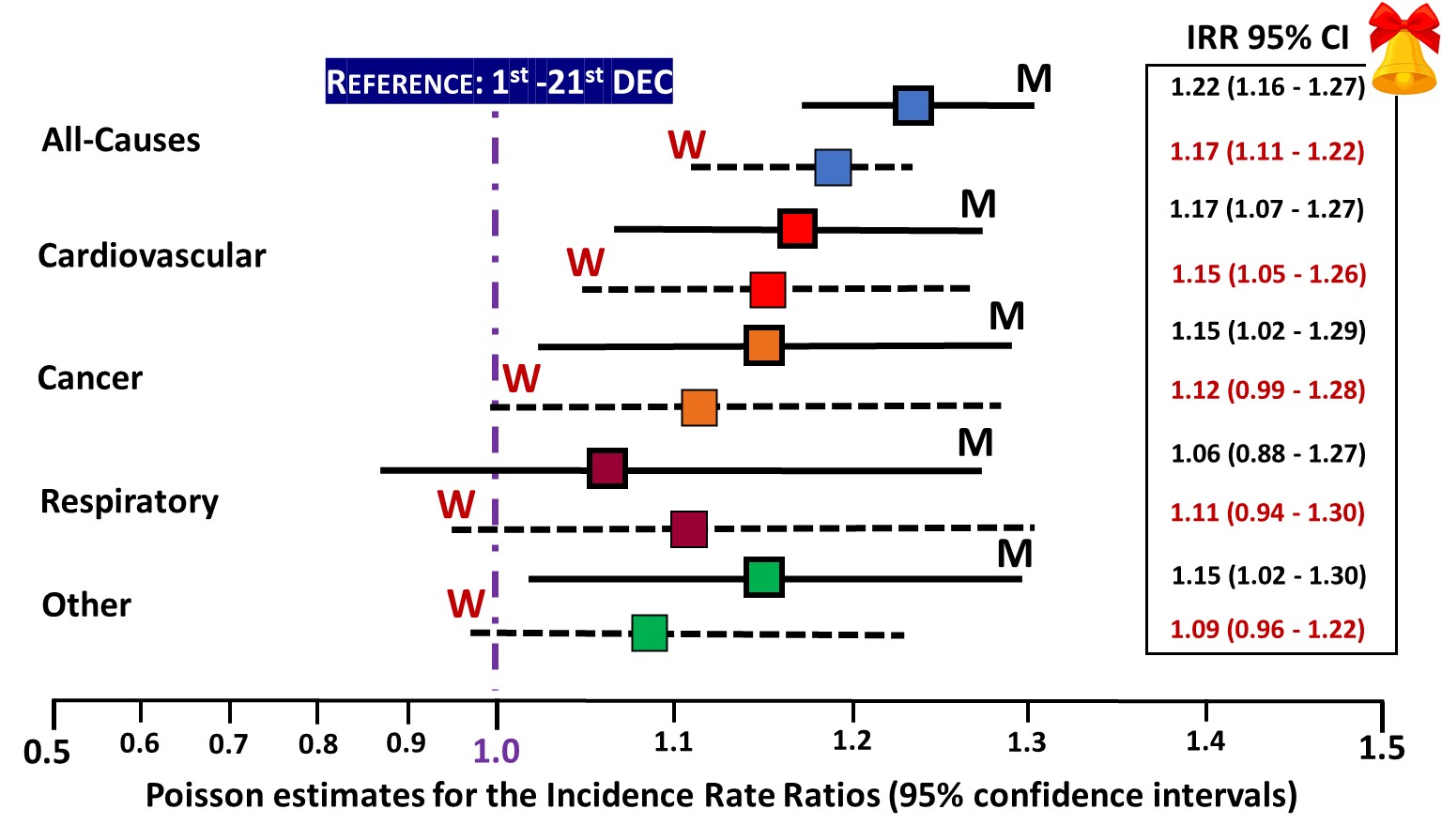
**

**Legend:**  Sex-specific differences in the incidence of daily mortality (all-cause and cause-specific) during December 22^nd^ – January 11^th^ compared to the reference 21-day period 1^st^ – 21st December. M=men (IRR and 95% CI in black) and W=women (IRR and 95% CI in red).

**Table S1 | Sensitivity analyses**

|  | **All-cause**  **mortality** | ***P***  **value** | **Cardiovascular**  **mortality** | ***P***  **value** | **Cancer**  **mortality** | ***P***  **value** | **Respiratory**  **mortality** | ***P***  **value** | **Other**  **causes** | ***P***  **value** |
| --- | --- | --- | --- | --- | --- | --- | --- | --- | --- | --- |
| **Model 1** | 0.30  (0.04 – 0.53) | 0.020 | 0.40  (0.11 - 0.62) | 0.005 | 0.07  (-0.19 - 0.33) | 0.586 | -0.03  (-0.23 - 0.16) | 0.740 | 0.06  (-0.19 - 0.31) | 0.648 |
| **Model 2** | 1.47  (1.27 – 1.66) | 0.000 | 0.95  (0.73 - 1.16) | 0.000 | 0.26  (0.04 - 0.47) | 0.020 | 0.09  (-0.07 - 0.24) | 0.267 | 0.26  (0.05 - 0.48) | 0.017 |
| **Model 3** | 0.43  (0.20 – 0.65) | 0.000 | 0.36  (0.12 - 0.59) | 0.003 | 0.23  (-0.003 - 0.472) | 0.053 | -0.16  (-0.36 - 0.04) | 0.118 | 0.05  (-0.20 - 0.29) | 0.708 |
| **Model 4** | 1.30  (0.99 – 1.61) | 0.000 | 0.87  (.57 – 1.16) | 0.000 | 0.13  (-0.17 - 0.42) | 0.395 | 0.02  (-0.24 - 028) | 0.870 | -0.28  (-0.61 - 0.04) | 0.088 |

Legend: In the first model, we used 3-days before Christmas (22^nd^-24^th^ December) and 3-days after Christmas (28^th^-30^th^ December) as the comparator. This model showed that daily all-cause and cardiovascular-related mortality during Christmas was significantly greater than the daily deaths recorded in any 3-days before or after Christmas. In model 2, we changed the comparator to any day in the calendar year except the Christmas period. Consistently, deaths during the 3-day Christmas window significantly exceeded deaths in any other day of the year. Model 3 compared Christmas deaths to death in any day of December except the Christmas window (comparator 1^st^-24^th^ and 28^th^-31^st^ December). Finally, we considered those baseline characteristics that could potentially influence the timing of death in our models to ascertain the impact of omitted variable bias on our estimates. The inclusion of these variables did not affect either the magnitude and statistical significance of estimated excess daily Christmas-related mortality for all-cause and cardiovascular-related mortality. Note: 95% confidence intervals are in parenthesis.
